# Flavonoid-rich foods (FRF): A promising nutraceutical approach against lifespan-shortening diseases

**DOI:** 10.22038/IJBMS.2019.35125.8353

**Published:** 2020-02

**Authors:** Alhamzah Hasan Waheed Janabi, Asghar Ali Kamboh, Muhammad Saeed, Lu Xiaoyu, Jannat BiBi, Fatima Majeed, Muhammad Naveed, Muhammad Jameel Mughal, Nazar Ali Korejo, Rubina Kamboh, Mahmoud Alagawany, Huixia Lv

**Affiliations:** 1 State Key Laboratory of Natural Medicines, School of Pharmacy, China Pharmaceutical University, Nanjing 211198, Jiangsu Province, PR China; 2 Department of Veterinary Microbiology, Faculty of Animal Husbandry and Veterinary Sciences, Sindh Agriculture University Tandojam 70060, Pakistan; 3 Faculty of Animal Production and Technology, Cholistan University of Veterinary and Animal Sciences, Bahawalpur 6300, Pakistan; 4 Department of Physical Education, Shaanxi Normal University, Xian, Shaanxi Province, P.R China; 5 School of Public Health, Nanjing Medical University, Jiangsu Province, 211166, P.R China; 6 School of Pharmacy, Nanjing Medical University, Jiangsu Province, 211166, P.R China; 7 Faculty of Health Sciences, University of Macau, Avenida de UniversidadeTaipa, Macau; 8 Department of Veterinary Medicine, Faculty of Animal Husbandry and Veterinary Science, Sindh Agriculture University, Tandojam 70060, Pakistan; 9 Liaquat University of Medical and Health Sciences, Jamshoro, Pakistan; 10 Departmentof Poultry, Faculty of Agriculture, Zagazig University, Zagazig 44511, Egypt

**Keywords:** Anti-oxidants, Cancers geroprotective, Cardiovascular diseases, Diabetes, Dietary supplements, Nutraceuticals

## Abstract

It is well documented that life expectancy in developed countries at birth is going to surpass the 20^th^ century. However, regrettably, a potential decline in life expectancy has been proposed for these nations in the 21st century due to a rapid upsurge in the prevalence of fatal degenerative diseases like cardiovascular diseases (CVD), cancer and diabetes. Collectively, these three diseases accounted for 65% of all deaths in urbanized societies and were considered as a dynamic issue for shortening the genetically determined lifespan through increased mortalities, morbidities, disabilities, immense sufferings, and premature aging. These fatal degenerative diseases and premature aging are closely associated with oxidative stress produced by the free radicals in the body. In epidemiologic studies, flavonoid-rich foods (FRF) like fruits, vegetables, and beverages have been associated as protective agents against these diseases. These also have been observed for their geroprotective effects and help in preventing premature aging and deterioration of brain function, which is related to Alzheimer’s disease and dementia. In this review, we presented a comprehensive overview of the FRF for their potential role against lifespan-shortening complications, i.e., CVD, cancer, and diabetes. We also have drawn the future perspective and dietary guidelines to reduce the fatal disease burden in urban populations.

## Introduction

Food concept in the world is changing from a past emphasis on hunger satisfaction, the absence of the classical nutrient deficiency diseases and survival to an increasing focus on foods promising use as functional ingredients to provide better well-being and health (1). Substantial data from epidemiological surveys and nutritional intervention studies indicate that different components of food have biological characteristics that exhibit activities in modulating animal and human metabolism in a manner favorable for the longevity and several fatal disease prevention (2-4), and foods containing these components are called “functional foods”, which are sometimes dubbed as ‘superfoods’ in medical communities (5, 6). The term “superfood” has been valuable for marketing purpose, having no precise scientific definition (6). Nevertheless, it is rising in the scientific literature with the sense of plant foods that contain phytochemicals that confer remarkable health benefits thus potentially increasing life expectancy. Until recently, the relationship between diet and lifespan has been hardly understood. Nevertheless, animal studies have revealed that dietary manipulation could extend the mean and maximum lifespan and significantly slow down the progression or even completely prevent the age-dependent pathologies (7). 

It is well documented that life expectancy at birth in developed countries has been increasing by three months per year since 1850 (8). Life expectancy at birth was 47.3 years at the beginning of the 20^th ^century, but now after a dramatic increase in the past 100 years, it is nearly 77 years, because of reduced mortalities from infections, increased income of people, and several other dietary, lifestyle-, and health-related factors (6, 7, 9). Regrettably, in the 21^st^ century, a potential decline in life expectancy has been proposed for developed nations (10) due to rapid trends in the prevalence of fatal diseases like cardiovascular diseases, cancer, and diabetes (10, 11), which accounted for 65% of all deaths in the USA. These diseases shorten life expectancy through increased mortalities, disabilities, and enormous sufferings and undermine health conditions (11). There is growing evidence that oxidative stress produced by some reactive oxygen compounds as well as free radicals such as nitric oxide (*NO) radical, hydroxyl (*OH) radical, lipid peroxyl (LOO*) radical, and superoxide (O_-2_) radical are the causative agents for a number of human diseases (12, 13). Therefore, particular attention is given to search for powerful antioxidant agents to attenuate the incidence and progression of lifespan-shortening diseases and to reinforce the genetically determined lifespan (13). 

Among natural antioxidants, phytochemicals which are ubiquitous in plants with their major nutraceutical part called flavonoids (14), are considered as potent natural antioxidants. Over 8000, various natural flavonoids have been already described as mentioned by Croft (15), and this list is still growing. Some health-oriented biological effects are attributed to their antioxidant potency (16). Flavonoids have been shown to possess antioxidant properties within *in vitro* experimental systems (4) and anti-inflammatory (17), immune-modulator (16), antiviral (18), antiallergic (19), and anticarcinogenic properties (20). Their antioxidant and inflammatory properties help in toxin-mediated stress and chronic disease prevention (21). Due to their surprising biological health effects, flavonoids are considered ‘disease-preventing, health-promoting dietary supplements’ (22). So, more than 30000 publications/ year in the past few years were focused on health-promoting impacts of flavonoids (23). Foods containing these flavonoids have also been attracting considerable attention in the medical and public communities because of evidence from various literature which suggest that consumption of Flavonoid-rich foods (FRF) could potentially improve human health and well-being (24). Numerous dietary intervention studies have proven that consumption of plant products (e.g., fruits and vegetables) declines the development of pathological condition risks, which include genetic and nervous system disorders, inﬂammatory and cardiovascular diseases, and cancer (25, 26). A series of studies in China indicated that high intake of fruits and vegetables could prevent breast cancer (27), coronary heart disease (28), cataracts, diabetes, Alzheimer’s disease, and even asthma (29, 30). According to the world health report, 4.4% of the overall disease burden including disability and mortality in Europe could be attributed to low fruit and vegetable intake (31). 

All these studies revealed that plant-derived FRF could reinforce life expectancy by cutting or preventing the risk of chronic lifespan-shortening disorders including diabetes, cancer, and cardiovascular diseases (10, 11) by dropping their associated risk factors, which positively affect the lifespan (32). The present review has focused on the evidence and mechanisms involved in the protective role of FRF against global lifespan-shortening complications including cardiovascular diseases, cancer, and diabetes. We also have drawn the evidence-based dietary guidelines for the readers to minimize the effects of aging, reduce the occurrence of degenerative diseases, and to get the genetically determined lifespan. Moreover, we have proposed a future perspective to identify priorities in food for a particular condition. 

**Sources and phytochemistry**

**Figure 1:**
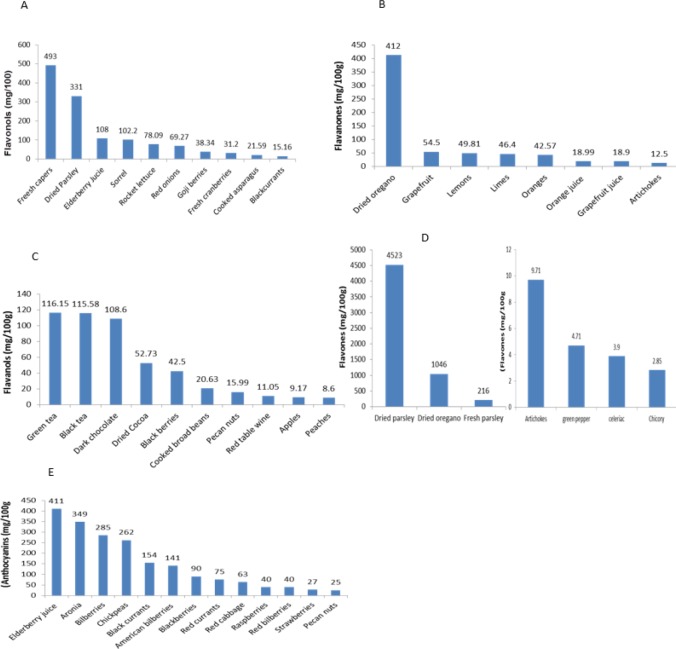
A. Mean concentration of flavonols (mg/100 g) in different food sources. B. Mean concentration of flavanones (mg/100 g) in various food sources. C. Mean concentration of flavanols (mg/100 g) in different food sources. For green and black tea, leaves to water ratio are 1:20 (w/v). D. Mean concentration of anthocyanins (mg/100 g) in different food sources E. Mean concentration of flavones (mg/100 g) in various food sources (37)

Flavonoid-rich foods, based on their surprising health effects are well described as superfoods. These include all plant origin foods mainly tea, fruit, vegetables, grains, legumes, nuts, and wine (33). Tea and wine are the primary dietary sources of flavonoids in eastern and western societies, respectively. Besides, leafy vegetables, onions, apples, berries, cherries, soybeans, and citrus fruits are considered an important source of dietary flavonoids (34-36). The content of flavonoids in different food sources is shown in Figures 1, A-E.

**Figure 2:**
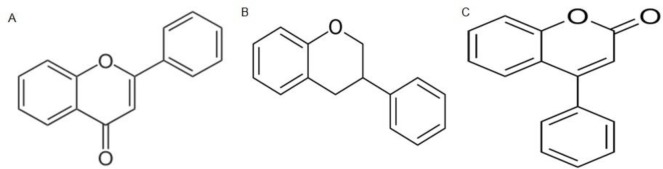
Basic chemical structural and types of flavonoids. A: Molecular structure of flavones backbone (2-phenyl-1,4-benzopyrene). B & C: isoflavan and neoflavonoids structures

The chemical structure of flavonoids consists of a 15-carbon skeleton, which contains two phenyl (A & B) rings and (C) heterocyclic ring. The carbon skeleton can be abbreviated C6-C3-C6. According to the IUPAC nomenclature, they can be categorized into three categories: flavonoids or bioflavonoids, isoflavonoids (3-phenyl-1,4-benzopyrone), and neoflavonoids (4-phenyl-1,2-benzopyrone) (33, 34). Flavonoids could be further classified into flavonols (e.g., quercetin, rutin), flavanones (e.g., naringenin, hesperidin), flavanols (e.g., epicatechin, gallocatechin), flavones (e.g., luteolin, apigenin), and anthocyanins (e.g., pelargonidin, malvidin). The typical examples of isoflavonoids (also called isoflavone) are genistein and daidzein; whereas, neoflavonoids occur in the form of isoflavones and are rarely found in plant foods (24). The chemical structure and types of flavonoids are shown in Figure 2.

**Mechanism of action of FRF**

Significant work has been conducted in recent years to explore the working mechanisms by which FRF exert their effects against pathological conditions. Most of the investigations have communicated that beneficial activity of these foods from anti-mutagenicity to anti-aging are attributed to antioxidant potency of flavonoids (38, 39). These flavonoids exert their anti-oxidative action in several ways including direct trapping and scavenging of free radicals, decreasing leukocyte immobilization, and regulation of nitric oxide and xanthine oxidase activity (40, 41). Several flavonoids, including quercetin, have been reported for the reduction in ischemia-reperfusion injury by interfering with inducible nitric-oxide synthase activity (42). Nitric oxide itself can be viewed as a radical, and it was reported that nitric oxide molecules are directly scavenged by flavonoids. Therefore, it has been speculated that nitric oxide scavenging plays a role in the therapeutic effects of flavonoids (41). The significant effects of flavonoids are due to radical scavenging but another possible mechanism by which flavonoids act is through interaction with various enzyme systems such as superoxide dismutase, catalase, and glutathione peroxidase (41). Furthermore, *in vitro* studies have declared the anti-proliferative activity of flavonoids through inhibition of polyamine biosynthesis and signal transduction enzymes like protein tyrosine kinase (PTK), protein kinase C (PKC) and phosphoinositide 3-kinases (PI3K), induction of apoptosis and cell cycle arrest at G1/G2, differentiation of transformed cells, and rehabilitation of cellular homeostasis (43, 44).

**Pharmacokinetics of flavonoids**

**Figure 3:**
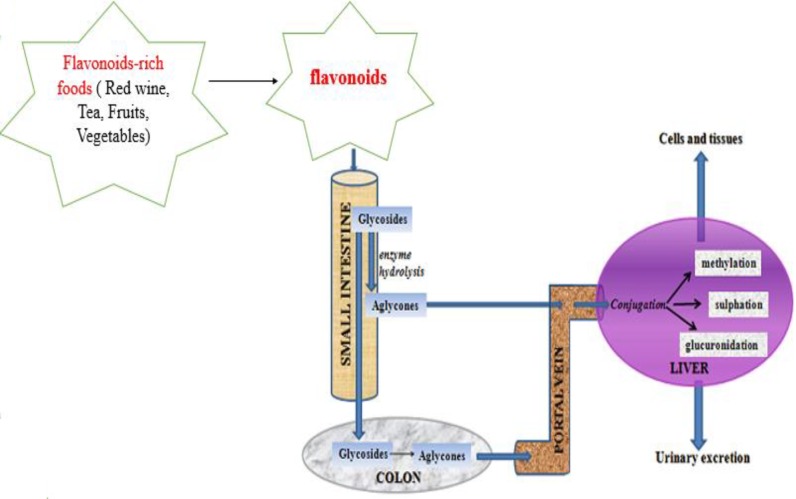
Pharmacokinetics of dietary flavonoids

The absorption, distribution, and biotransformation of flavonoids from FRF are crucial for their biological effects against lifespan-shortening degenerative diseases as shown in Figure 3. The majority of flavonoids found in FRF are in the form of β-glycosides, and hydrolysis of the glycoside moiety is an essential step for absorption. Hydrolysis occurs in the cecum and colon by enterobacteria, aglycones are absorbed by gut epithelial cells and entered the circulation to metabolize in the liver (45). It is also reported that lactase-phlorizin hydrolase (LPH, EC 3.2.1.62), which is found on the brush border of the mammalian small intestine for the breakdown of lactose, is responsible for hydrolysis of flavonoids (46). By using healthy ileostomy volunteers in an experiment, it is demonstrated that sodium-dependent glucose transporter-1 (SGLT-1) is responsible for the transport of quercetin glucosides from intestinal epithelial cells (46).

Conversely, in another experiment, it was claimed that quercetin glucosides are entirely hydrolyzed to their aglycone form before transport from the intestinal tract in ileostomy patients (47). Overall, the intestinal absorption, distribution, and metabolism of flavonoids are not well elucidated at present; thus, the events in the intestinal tract should be clarified to observe the desirable health effects of FRF. Moreover, research on the mechanisms for aglycone transfer across the gut wall need to be illuminated.

It has been observed that bioavailability of flavonoids is very low, i.e., 2 –20% on an intake of FRF like vegetables, fruit juices, and beverages (48). Therefore, this area of research needs more attention to find possible ways for enhancement of flavonoids absorption. 

Food matrix also plays a vital role in the bioavailability and pharmacokinetics of flavonoids from FRF. A liquid matrix yields a faster absorption rate and higher peak plasma concentrations than a solid matrix, whereas aglycones in fermented foods are absorbed more rapidly than glucoside conjugates (49). Dietary fat (3–5 g/meal) is also reported as an enhancer for the absorption of phytochemicals from vegetables in children (50).

It is also well established that processing (mechanical or heat treatment) could increase the biological activity of FRF by promoting the bioaccessibility of polyphenols in food matrix (51). Tomato polyphenols (e.g., naringenin) are trapped in the cutin matrix of the membrane of the ripe fruit where it strongly interacts with insoluble polyesters. Mechanical and heat treatments may break the interactions, thus improving flavonoids bioaccessibility *in vivo *(52). Nevertheless, a few studies have reported the adverse effect of processing on garlic’s ability to alter the bioactivation of a known experimental mammary carcinogen (53). This might indicate the different response of each FRF to processing. Thus, studies are needed to unravel the individual response of processing for all FRF with significant insight into processing time. Some creative tools are required to explore the exact relationship of FRF with other components of diet and food matrix to make their possible synergisms and to avoid antagonistic combinations to enhance their bio-functionality against lifespan-shortening complications.

**Pharmacological values and therapeutic properties**

**Figure 4:**
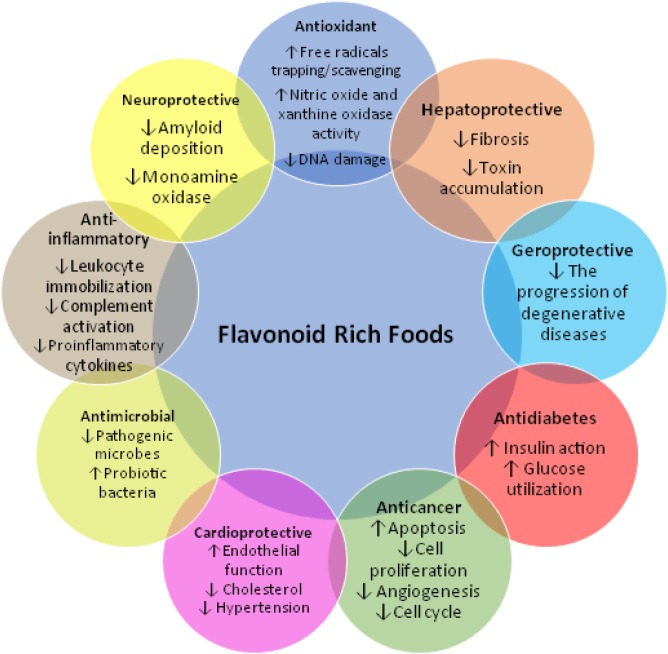
The schematic overview of the therapeutic properties of flavonoid-rich foods

At the end of the 20^th ^century, it was concluded for the first time that traditional Mediterranean diet meets several important criteria (Figure 4), which is responsible for extended lifespan (54) and low incidence of fatal degenerative diseases like cardiovascular diseases, cancer, and diabetes (55, 56). This conclusion was drawn by intriguing evidence accumulated over the last three decades that supported the Mediterranean diet having a high amount of FRF, i.e., olive oil, legumes, cereals, vegetables, and fruits for these effects. In Greece, in addition to high vegetable diet, the wild edible greens are eaten frequently in the form of pies and salads and contain a very high concentration of flavonoids (more than those in torn or wine) as reported by a study (54). The antioxidants found in these foods (FRF) such as polyphenolic flavonoids correct the free radicals generated in all cells from normal oxidative reactions, which if left uncorrected may damage cellular proteins, lipids, and nucleic acids, resulting in the onset of lifespan-shortening diseases (Table 1).

atal degenerative diseases including CVD, cancer, and diabetes are the primary health burdens, which reduce the average life expectancy and impair the health status. CVD is the leading cause of death followed by cancer, and both together account for almost one-half of all deaths (9) whereas, collectively all three (cardiovascular diseases, cancer, and diabetes) account for nearly two of every three persons in the USA and comprise 32% health cost of total illness costs (11). A summary of studies indicating the effects of FRF in reducing mortalities have been summarized in Table 2.

***Protective effect against diabetes***

 Diabetes is an epidemic health concern in both developed and developing countries, characterized by impaired insulin production and function. In the United States, 7.8% of the population have diabetes, 35.4% have impaired fasting glucose (IFG) levels, 15.4% have impaired glucose tolerance (IGT), and 40.1% have pre-diabetes (IFG, IGT, or both) (57), while in China 9.7% and 15.5% of people are positive for diabetes and pre-diabetes, respectively (58). Diabetes could reduce life expectancy up to 15 years and account as a significant cause of mortalities in both developed and developing countries (59). It is also associated with other chronic diseases like stroke, heart diseases, nervous system disorders, kidney diseases, and vision problems. Its prevention is more accessible than a cure. In randomized trials, it was consistently observed that increased physical activity and dietary manipulation are the ideal approaches to prevent diabetes (56).

Numerous physiological studies had declared that free radicals might contribute to the autoimmune destruction of pancreatic β cells, leading to diabetes (60), and may impair insulin action (61). Fruits, vegetables, and whole grains possess strong scavenging ability against these radicals resulting in reduced risk of type I and type II diabetes mellitus (62, 63). It was also hypothesized that plant-derived foods like fruits and vegetables are low in carbohydrate contents, therefore could prevent the rise of blood sugars (56). Other dietary factors that have been related to reducing the risk of type II diabetes include coffee (64), berries (63), and tea (65). High intakes of quercetin and myricetin, mainly from dietary consumption of apples and berries also associated with reduced risk of type 2 diabetes (20). Among European adult persons who drank coffee frequently (≥7 cups/d) had a 29% to 52% reduced risk for diabetes compared with those who drank less coffee (≤2 cups/d or no cups/d) (64, 66). Moreover, in another study in Japan, it was reported that consumption of green tea, coffee, and large total caffeine was associated with a reduced risk for type 2 diabetes (67).

**Figure 5:**
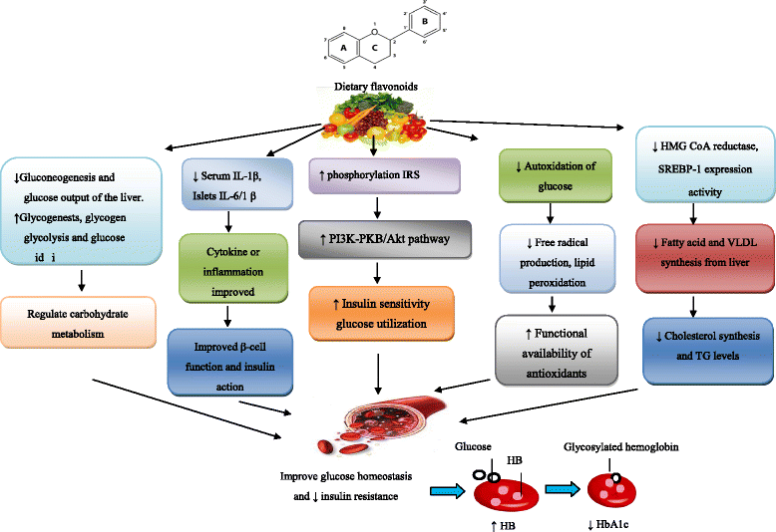
The proposed antidiabetic effects of flavonoids foods. ↑ increase; ↓ decrease; AKT: v-akt murine thymoma viral oncogene homolog; HB: hemoglobin; IRS: insulin receptor substrate; HbA1c: glycated hemoglobin; IL-1β: interleukin-1 beta; HMG-CoA: 3-hydroxy-3-methyl glutaryl-coenzyme A; PI3K: phosphatidylinositol-3-kinase; TG: triglycerides; SREBP-1c: sterol regulatory element-binding protein; VLDL: very low density Lipoprotein (71)

Furthermore, antidiabetic (blood glucose reducing) effect of green tea (68) and black tea (69) has also been observed, indicating them as potent preventive and curative agents. Onion and garlic also have been found to have antihyperglycemic effects (70), probably based on their phytochemical contents. The antidiabetic results of FRF have been summarized in Figure 5.

***Protective effect against cancer***

**Figure 6:**
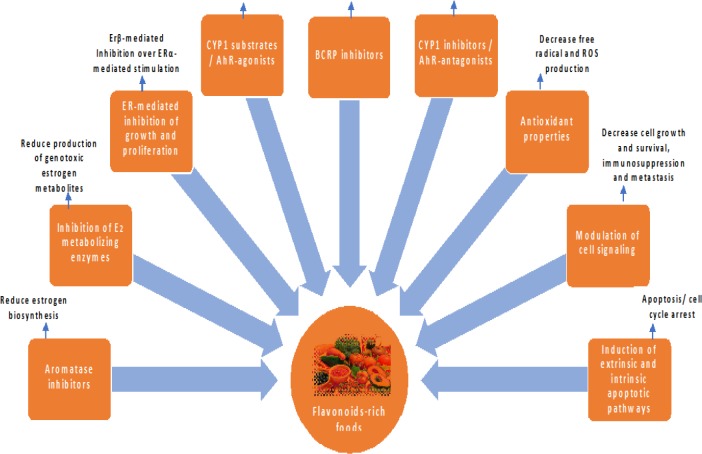
The schematic overview of the anticancer effects of flavonoid-rich foods. CYP1: cytochrome P450; BCRP: breast cancer resistance protein; AhR: aryl hydrocarbon receptor; Erβ: estrogen receptor β; ROS: reactive oxygen species

The oxidative stress from exposure to industrial chemicals, air pollutants, ionizing radiation, or ultraviolet light, might overwhelm the antioxidant system of the body and cause oxidative damage of nuclear acids and proteins, which leads to cancer initiation in addition to other degenerative diseases as explained by another study (13). Until now, no data are available assuring anticarcinogenic impacts of foods in humans. However, there are many rodent* in vivo* and *in vitro* studies confirming chemopreventive impact of certain plant foods against early stages of cancer (72). Epidemiological studies are also supporting FRF for their cancer preventing effects particularly, cruciferous vegetables due to the abundant presence of anticarcinogenic compounds such as polyphenols and isothiocyanates (73, 74). Verhoeven *et al.* (75) claimed inverse associations between intake of crucifer and the incidence of skin, pancreas, lung, prostate, bladder, stomach, colon, and thyroid cancers. In case-control studies, inverse associations between risk of cancer and intakes of broccoli, cauliflower, brussels sprouts, or cabbage were noted in 70%, 56%, 67%, and 29%, respectively (76). A Finnish study specified that risk of prostate cancer lowered at higher intakes of myricetin (from berries), and risk of breast cancer lowered at higher intakes of quercetin (from apples and onions) (20). Soybean consumption is demonstrated as a contributing factor in lowering prostate and breast cancer in Japanese men and women, respectively. This effect is considered due to isoflavone genistein that functions as an estrogen antagonist and reduces the risk of estrogen-sensitive tumors (77). Tomato and tomato products are also being investigated for their significant role in cancer chemoprevention, especially in prostate cancer (78). In another animal study, the chemopreventive effect of tea was observed for various types of cancers and recognized for the antioxidant property of polyphenolic components known as catechins (79). Among spices, chemopreventive effects of turmeric against cancers of the skin, mouth, fore stomach, liver, and colon are well documented (80, 81). These spices contain several natural water-soluble phenolic acids and flavonoids, such as caffeic acid and quercetin that attribute to these effects by inhibition of procarcinogen activators or induction of carcinogen deactivation enzymes (72). The National Cancer Institute, after five years of research, revealed the anticancer potential of plant foods. The foods and herbs with the highest anticancer activity include garlic, soybeans, cabbage, ginger, carrots, celery, cilantro, parsley, and parsnips, while those with a modest level of cancer-protective activity include onions, citrus, turmeric, cruciferous vegetables (broccoli, brussels sprouts, cabbage, and cauliflower), tomatoes, peppers, and whole wheat (82) and their anticancer effects have been summarized in Figure 6.

***Estrogenic effect***

Some dietary flavonoids (including isoflavones and prenylflavonoids) are known as phytoestrogens, which can interact with estrogen receptors (ER) or modulate estrogen action *in vivo*. These phytoestrogens (e.g., genistein) are non-steroidal in chemical structure, but due to the presence of phenolic rings, particularly the 4’-hydroxyl, they can bind estrogen receptors. At specific concentrations, which may depend on many factors including receptor numbers, occupancy, and competing for estrogen concentration, they may antagonize and inhibit estrogen action (83).

There are inconsistent reports for the effects of phytoestrogens found in FRF on breast cancer. Setchell (83) demonstrated that soy isoflavones could stimulate the growth of ER-positive breast cancer cells through the ER singling pathway; while, some other studies stated the putative effect of isoflavones to inhibit carcinogenesis (84, 85). These chemopreventive effects may be through impairment of protein tyrosine kinases (86) or topoisomerase II inhibition (87). From the reports in support of the positive and negative effects of phytoestrogens on breast cancer, it is indicated that more clinical studies are warranted to clarify this critical issue. Other factors like hormonal status, age, the timing of exposure, and individual metabolism should also be considered. At this stage, recommendation of nutritionists to avoid soy foods is not logical (83), and the use of phytestrogens as supplements will be entirely acquitted.

***Protective effect against cardiovascular diseases***

Potential health benefits of chocolate (88) and tea (89) regarding heart health have been well-documented and attributed to ﬂavonoid procyanidins, which reduce low-density lipoprotein (LDL) cholesterol by 11.1%. This LDL is oxidized directly by peroxynitrite, which is produced by the reaction of nitric oxide with free radicals. The nitric oxide release through the constitutive nitric oxide synthase activity is essential in the maintenance of blood vessels dilation (40). Flavonoids are capable of affecting different cells included in atherosclerosis development, one of the leading reasons for cardiovascular diseases. The chemokine monocyte chemotactic protein 1 (MCP-1) is well known to mediate macrophage recruitment to infection or inﬂammation sites, and direct involvement of MCP-1 on atherogenesis has been established. Furthermore, flavonoids could inhibit aggregation of TRAP-induced platelet (90) and protect endothelial cells from CD40-induced pro-inﬂammatory signaling as found by a study (91). 

**Figure 7:**
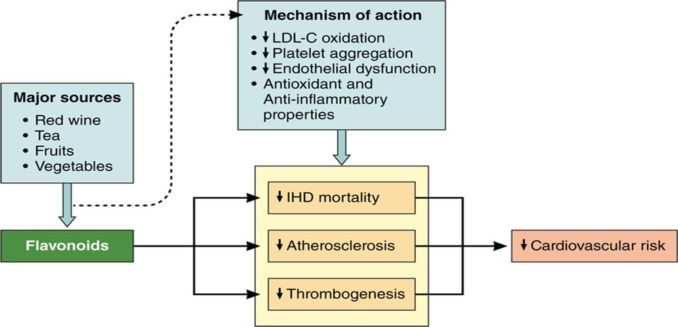
The cardioprotective effect and implicated mechanisms of flavonoids. (IHD) indicates ischemic heart disease and (LDL), low-density lipoprotein cholesterol (92)

Several epidemiological studies proved that diets rich in speciﬁc antioxidants from fruits, some vegetable oils, and vegetables diminish the relative risk of premature death from CVD (25, 92) as shown in Figure 7. Findings of observational population studies support the hypothesis that fruit and vegetable consumption have a regulating effect on hypertension (93), hypercholesterolemia (94), and obesity (95), which are important cardiovascular risk factors. In a study related to Dietary Approaches to Stop Hypertension (DASH), it was concluded that intake of 1-2 cups of vegetable juice daily is associated with a reduction of blood pressure in subjects who were prehypertensive at the start of the trial (96). Furthermore, the decreasing trend for ischemic heart disease mortality was observed with the consumption of apples and onions (20). These findings are consistent with a large prospective study of postmenopausal women with 16 years follow-up that indicated dietary intakes of apples, pears, and red wine were associated with a lower risk of all-cause mortality, death due to coronary heart disease (CHD), and death due to CVD (36). In a cohort study of about 100,000 people, it was concluded that five servings of fruits and vegetables daily cause 28% reduction in risk of cardiovascular diseases (97), which is also in agreement with American Heart Association (AHA) recommendation for consuming at least five servings of fruits and vegetables per day (98).

**FRF as geroprotective**

Aging is an essential part of life, which is directly proportional to lifespan. It may not be eliminated from life, but it could be potentially accelerated or decelerated (99). Target health issues related to the aging process include the process of oxidation, the promotion of bone health, memory retention and cognition. Decrements in motor function and memory are two main behavioral parameters that altered senescence in both humans and animals; however, they appear due to increased amounts of inflammatory markers and/or enhanced susceptibility to oxidative stress caused by reactive species from oxygen and nitrogen and subsequent induction of peroxidative reactions that result in damage to biomolecules (100). 

The potential substances that can slow down the aging process, prevent premature aging, and increase life expectancy, are known as geroprotectors (99). Natural antioxidants such as flavonoids have been observed as efficient geroprotectors and lifespan extending compounds through down-regulating the progression of degenerative diseases (101), and are also called lifespan-essential ingredients (102). Therefore, the use of FRF with strong antioxidant potential may reduce age-related disorders. This may be achieved by scavenging damaging ROS, preventing the formation of lipid peroxides, protecting proteins and DNA from oxidative damage, decreasing inﬂammation, and protecting against ROS-mediated apoptosis (103). In addition to free radical scavenging, anti-inflammatory role of flavonoids is also crucial for overreaction of microglial cells for signals thus reducing the production of cytokines causing behavioral pathology, including cognition and restore the population of microglial brain cells to put the elder brain in the youthful state (104). It was also observed that these FRF could directly alter the neuronal communication, calcium buffering, neuroprotective stress shock proteins, and stress signaling pathways for the amelioration of age-related deficits (105). 

The role of diet in minimizing the adverse effects of aging has been extensively investigated. It has been concluded that nutritional interventions, via the polyphenolics present in plant foods like fruits and vegetables, may correct the age deficits and age-related deterioration of brain function (17, 106). Furthermore, Joseph and coworkers expressed that anthocyanin-rich fruits such as blueberry, spinach, and strawberry may help reverse the course of neuronal and behavioral aging (107). Moreover, by using transgenic mice as a model for Alzheimer’s disease, the same group reported the beneﬁcial effect of blueberry extracts on the outcome of this neurodegenerative illness (108). Studies also suggested the antiaging and brain protective role of garlic linked to dementia and Alzheimer’s disease (103). In another study, green tea and its flavonoids constituents were proven for efficacy as prophylactic and neuroprotective agents against age-related neurodegenerative and neuroinflammatory diseases such as Parkinson’s disease and multiple sclerosis (109). The study also confirmed that (-)-Epigallocatechin gallate inhibits lipopolysaccharide-induced microglial activation and protects against inflammation-mediated dopaminergic neuronal injury, Alzheimer’s disease via modulation of cell survival/death genes, and mitochondrial function, which contributes to neuronal viability (109). 

**How to get maximum health benefits from FRF**

Epidemiological and clinical evidence indicate that like essential nutrients of foods (e.g., vitamins), which are vital for several physiological and pathological conditions, flavonoids are necessary for full genetically-determined lifespan through down-regulation of chronic degenerative diseases (102). Therefore, it is essential to intake the optimum amount of flavonoids by including the reference quantity of FRF on the regular menu.

Flavonoid-rich green tea, isoflavone-rich soy, flax seed, ﬂavonol quercetin, and isoflavones are popular supplements among consumers. People are showing their interest in these supplements because they assume that they are not consuming sufficient quantities of dietary flavonoids and ﬂavonoids supplements are devoid of toxicity because these compounds are “natural.” However, there is evidence, which indicates the harmful effects of flavonoids supplements. In both animal and human studies, anti-thyroid and goitrogenic activities were observed by a high dose of green tea extracts and isoflavones (110, 111). Likewise, another adverse effect of high flavonoids doses includes inhibition of vitamin C transport, decreased trace element bioavailability, and impaired foliate uptake (112).

Feeling the gravity of this ambiguous situation it is advisable to use all flavonoid supplements with the guidelines of a health practitioner/nutritionist because these are providing hundreds of times higher doses than a regular diet and only use the optimum amounts of fresh fruits and vegetables. A large body of nutritionists, medical experts, and the American Heart Association (AHA) (97, 98) also suggests this. The instructions of regulatory bodies like WHO (world health organization), AHA, ACS (American cancer society), and ADA (American diabetes association) should strictly be followed for consumption of at least 5 servings of fresh fruits and vegetables, whole grains, legumes, flavonoid-rich beverages, and fruit juices to get the potential health benefits from these FRF. It is also acceptable to take a moderate amount of wines, meat, milk, and other dairy products to keep in mind the possible role/effects of these substances in the food matrix. A moderate amount of heart-healthy fat (proportional monounsaturated and polyunsaturated fats) should also be included in the dietary menu for its helping role in the absorption of FRF. To ensure the maximum health benefits of FRF, healthy lifestyle, including regular exercise, should also be adapted to maintain the active physiological status of the body.

***Biomarkers***

Intake biomarkers are useful for reflecting the amount of food or metabolites present in the body cells or fluids, but there is a need to know the best time for their measurement after consumption. Evidence is emerging that some biomarkers for cardiac health and other fatal degenerative diseases have been used successfully to differentiate disease and non-disease states and to predict the association between dietary intake and future susceptibility to infections (113, 114). For evaluation of the merits of nutritional habits, many genes, associated products, and receptors have been investigated for fatal degenerative and age-related diseases (like cancer, diabetes, Parkinson’s disease, etc.) as markers of genetic susceptibility including p53, PPAR-α, APOA1, OB, and BCL (115, 116). Overall, authentic intake and susceptibility biomarkers are probably needed to develop a profile for an individual to approach health and longevity and significantly reduce the incidence of diet-related diseases through improved management of disease and ultimately its prevention. However, the relationship between dietary intake and lifespan related biomarkers is often highly complex.

***Dietary/lifestyle patterns***

Dietary factors are seen as contributing to the leading causes of death of urban people, including CHD, diabetes, and certain types of cancers. Inappropriate dietary habits are responsible for poor health and reduced lifespan, which are also a major reasons for public interest in the use of alternative medicines and functional foods (117). Advances in nutritional genomics like development of the concept of gene-nutrient interaction gets significant emphasize from dietetics profession particularly the clinical dietitians to suggest appropriate foods to their patients. Unquestionably, dietary habits are not the sole determinant of disease states, because adjusting the dietary menu with FRF represents a significant way of reducing risk. Further studies are recommended to characterize the strength of diet–health association, its generalizability, the dose-response relationship, and the timing of diet for potential benefits. 

Some reviews have been published on the potential role of FRF in the prevention of cancer (73, 75) and other lifestyle-related diseases like hypertension, diabetes, obesity, and aging (118). However, there is much diversity in the cultural dietary patterns and lifestyles such as the degree of physical activity and consumption of low fat and low-calorie diet, which adds complexity in understanding the exact biological role of FRF in disease prevention. Therefore, more epidemiological studies are needed in different geographical locations to correlate the dietary patterns with cultural and individual habits and to minimize the wide divergence of the population. 

**DRI in terms of effective dosages**

Regrettably, dietary reference intake (DRI) for flavonoids could not be established until now due to insufficient data regarding flavonoid contents of different foods and variations in different varieties and cultivars and grown in different environmental conditions, production methods (organic/conventional), post-harvest processing, and storage effect on flavonoids concentration of foods (48). Another difficulty is the unavailability of authentic and certified means for determining the flavonoid contents of several foods (FRF). On the other hand, there is growing interest in developing dietary supplements containing flavonoids or flavonoid-rich foods. In such dietary supplements usually, active components (a mixture of polyphenols) from FRF were purified or concentrated to boost the antioxidant status of the consumer (119). Nevertheless, presently all dietary recommendations by scientific communities like AHA for consumption of five servings of fruits and vegetables per day are based on nutritional epidemiological surveys or *in vitro* studies but not supported by *in vivo* clinical literature. Therefore, there is a need to use the tools of food science and technology to establish a data bank for flavonoid contents of all foods with an estimated difference of biological and physical factors to determine the therapeutic/preventive dosages/amount of FRF for fatal degenerative diseases. Clinical trials are also necessary to know the relationship between specific FRF and lifespan-shortening disorders. Consequences of long-term intake of FRF should also be explored.

**Conclusion and future perspectives**

Dietary factors play a vital role in the development and preclusion of premature aging, and some fatal degenerative diseases ultimately shorten the genetically determined lifespan. These life-threatening neurodegenerative diseases and age-related metabolic disorders are closely associated with oxidative stress produced by free radicals in the body. It has been estimated that free radicals are involved in the etiology of several (>100) human diseases and the aging process. ased on their potent antioxidant properties, flavonoids protect against these diseases and could potentially modulate life expectancy. Clinical evidence supports the health promoting, disease preventing, and life-extending effects of FRF like fruits including citrus fruits, berries, apples, vegetables particularly deep-colored green vegetables and onion, beverages such as tea and red wine, and cocoa that make these foods approach the superfoods of the millennium. It is strongly recommended that consumers eat 5 to 10 servings (one serving: about 40 g) of a wide variety of fruits and vegetables daily to reduce the risk of fatal degenerative lifespan-shortening diseases and to meet the nutrient requirements for optimum lifespan. However, one should keep in mind that FRF are not “magic bullets” for disease-free long life (120). It is only one part of a comprehensive lifestyle, which should be adjoined by physical activity (at least 30 min/day), smoking avoidance, stress reduction, moderate consumption of alcohol, meat, eggs, and dairy products, low consumption of fat and sugars, maintaining healthy body weight (BMI: <25 kg/m^2^), maintenance of healthy environment, and other positive health practices. When all of these issues are addressed together, then FRF becomes part of an efficient strategy to maximize lifespan and cut the risk of lifespan-shortening degenerative diseases.

**Table 1:** Effects of flavonoids rich-foods against lifespan-shortening diseases

**Type of flavonoids rich-food**	**Biological and pharmacological effects**	**References**
Fruits/vegetables	Anti-hypertension, reduced risk of diabetes, anti-hypercholesterolemia, anti-obesity, ↓ cardiovascular diseases, ↓ breast cancer, ↓ coronary heart diseases	[27, 28, 62, 93, 94, 95, 97]
Whole grains	Reduced risk of diabetes, anticancer	[63, 82]
Coffee	Reduced risk of type 2 diabetes	[64]
Berries	↓Prostate cancer, reduced risk of type 2 diabetes	[20, 63]
Green tea	Reduced risk of type 2 diabetes, ↓ blood glucose, anticancer	[65, 68, 79]
Apple	Reduced risk of type 2 diabetes, ↓ breast cancer, ↓ cardiovascular diseases	[20, 36]
Black tea	↓Blood glucose, ↓ total and LDL cholesterol, ↓ myocardial infarction, reduced risk of coronary heart disease	[69, 89, 121]
Onion	Antihyperglycemic effects, ↓ breast cancer	[20, 70]
Garlic	Anti-platelet aggregation, modification of LDL, antihyperglycemic effects, anticancer	[70, 82]
Cruciferous vegetables	Anticancer	[73]
Cabbage	Anticancer, ↓ vascular diseases	[20, 76]
Broccoli	Anticancer, ↓ prostate cancer	[76]
Cauliflower	Anticancer, ↓ prostate cancer	[76]
Brussels sprouts	Anticancer	[76]
Soy	Reduced risk of breast and prostate cancer	[77]
Citrus fruits	Antiproliferative, ↓ vascular diseases	[20]
Tomato	↓ Prostate cancer	[78]
Turmeric	Anti-hepatocarcinogenesis, anticancer	[80, 81]
Ginger	Inhibit platelet aggregation, anticancer, anti-thrombotic	[82, 122]
Carrots	Anticancer	[82]
Pomegranate	Anticancer	[44]

↓: decreased; LDL: low-density lipoprotein

**Table 2:** Summary of studies indicating reduced mortalities by intake of flavonoids rich-foods (FRF)

**Type of FRF**	**Dose**	**Type of study**	**Number of participants**	**Follow-up period**	**Outcomes**	**References**
Fruit and vegetable	≥5 servings/day	Population-based cohort	71,706	13 year	53 percent higher all-cause mortality rate in those who never consumed fruits and vegetables than those who consumed five servings/day	[123]
Cruciferous vegetable	144, 232, 307, 398 & 583 g/day	Prospective cohort study	134,796	4.6 year	A dose-response pattern was evident for increasing quintiles of cruciferous vegetables intake and reduction in total mortality as well as CVD mortality	[124]
Fruit and vegetables	178.8, 316.8, 468.4 & 725.4 g/day	Population cohort study	451,151	10 year	A stronger association was observed for raw vegetable and fruit consumption and reduction in all-cause mortality as well as CVD mortality	[125]
Coffee	1 to >6 cups/day	Cohort study	41, 836	15 year	1-3 cups/d significantly reduced the mortality from CVD and other inflammatory diseases in postmenopausal women	[126]
Green tea	1 to >5 cups/day	A population-based, prospective cohort study	40, 530	11 year	A 16% lowered mortality from all-cause and CVD in people who consumed 5 or more cups/day than those consuming less than 1 cup/day	[127]
Fruit, vegetable, and beans	Fruit: 0.9, 2.3, 3.9 &5.9 servings/wk; Vegetable: 1.2, 2.3, 3.4 & 5.2 servings/wk; Bean: 0.8, 1.8, 3.0 & 4.5 servings/wk	Cohort study	59,485	13 year	Fruit intake followed by vegetable and bean intake exhibited the significant inverse association for total and CVD mortality	[128]
Whole grains	≥3 servings/ day	Cross-sectional study	535	3 year	A significant inverse association was recorded for whole-grain intake and mortality from CVD	[129]
Green-yellow vegetables and fruits	≤ 1, 2-4 servings/wk and one serving daily	Prospective study	38, 540	18 year	Subjects had 12% lower mortality from all cancers and 20% lower mortality from lung and stomach cancer; as well as 8% lower mortality from all cancers and 25% lower mortality from liver cancer, those consuming 1 or about 1 serving/day of fruits and green-yellow vegetables, respectively, comparing with those who ate these foods once per week or less.	[130]
Fruit and vegetables	0.87, 1.61, 2.31, 3.21 & 4.89 servings/day	Prospective study	6,151	13 year	Compared with the bottom fifth, highest fifth of fruit and vegetable consumers had a significantly lower all-cause, cancer, and CVD mortality	[131]
Fruit and vegetables	< 5 to ≥5 servings/d	Cohort study	501	18 year	Individuals consumed the combination of ≥5 servings/day of fruits and vegetables and ≤12 energy from saturated fat had lower all-cause (31%) and CVD (76%) mortality, compared with those consuming <5 servings/day of fruits and vegetables and >12% saturated fats	[132]
Fruit and vegetables	<1 to 8 times/day	Cohort study	9,608	19 year	Fruit and vegetable consumption ≥3 times/day as compared with <1 time/day significantly reduce stroke mortality (42%), ischemic heart disease mortality (24%), CVD mortality (27%), and all-cause mortality (15%)	[133]
Fruits	0-1, 2-3, 4-5, 6-7 times/wk	Prospective cohort study	792	26 year	Significantly lower total and CVD mortality were recorded in men with high fruit consumption. This association existed up to 16 year follow-up (at the age of 70)	[134]
Fruit, berry, and vegetables	<133, 133-214, 215-293, 294-408 &>408 g/day	Prospective cohort study	2,641	12.8 year	A significantly lower CVD, non-CVD, and all-cause mortality was observed in men with the highest consumption of fruit, vegetable, and berries	[135]

## Conflicts of Interest

The authors declare that there are no conflicts of interest. All authors read and approved the final manuscript.
